# Association of change in physical activity with use of outpatient specialist care and hospitalisations among breast cancer survivors with type 2 diabetes in Sweden

**DOI:** 10.1038/s41416-025-03099-x

**Published:** 2025-07-04

**Authors:** Genevieve Allen, Emerald G. Heiland, Stanley Teleka, Ingrid Glimelius, Karl Michaëlsson, Liisa Byberg, Hannah L. Brooke

**Affiliations:** 1 Medical Epidemiology, Department of Surgical Sciences, Uppsala University, Uppsala, Sweden; 2 Department of Clinical Sciences, Lund University, Malmö, Sweden; 3 Department of Immunology, Genetics and Pathology, Unit of Cancer Precision Medicine, Uppsala University, Uppsala, Sweden

**Keywords:** Breast cancer, Type 2 diabetes, Epidemiology, Breast cancer, Quality of life

## Abstract

**Background:**

Studies in breast cancer patients have consistently shown that physical activity improves survival. We examined if change in physical activity from before to after a breast cancer diagnosis is associated with outpatient specialist care use and hospitalisations in women with type 2 diabetes mellitus (T2DM).

**Methods:**

This register-based cohort study included 2145 women with T2DM who self-reported frequency of walking 30 min/week, 1–3 years before and after their breast cancer diagnosis (diagnosed 2004-2018). Women were grouped as: maintained inactive (reference), increased activity, decreased activity, or maintained active. Using multivariable-adjusted Cox proportional hazards models, we assessed change in physical activity with time to 1) first hospital admission and 2) first outpatient specialist care/hospitalisation for 15 International Classification of Disease chapters in the National Patient Register.

**Results:**

Women who maintained active (hazard ratio[95% CI]: 0.68[0.58–0.80]) or increased (0.72[0.58–0.88]) activity, but not those who decreased (0.92[0.79–1.08]) activity, had a lower rate of hospitalisations during follow-up than inactive women. Results were similar for infection, malignancy, blood disorders, general symptoms, mental/behavioural health.

**Conclusion:**

In breast cancer survivors, treated with curative intention, with T2DM, maintaining or increasing walking to ≥3times/week was associated with lower rates of hospitalisations overall and specialist outpatient care/hospitalisations across a range of diagnoses.

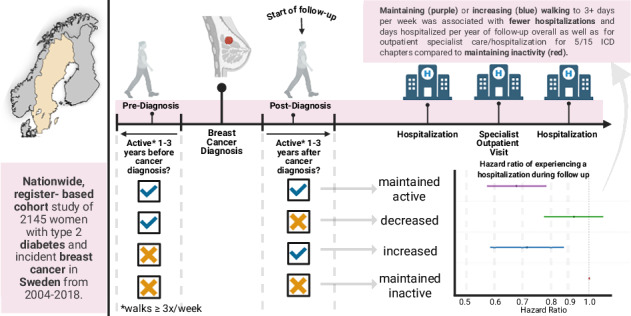

## Introduction

As breast cancer risk increases with age and excess weight, many women with breast cancer have previously been diagnosed with comorbid conditions such as type two diabetes (T2DM). Due to the prevalence of both T2DM and breast cancer, as well as emerging data indicating that T2DM is causally associated with worse breast cancer-specific survival [1], patients who concurrently have both conditions merit a dedicated research focus.

Knowledge about the change in physical activity from before to after a cancer diagnosis is increasingly recognised as an essential area of research as it incorporates information on both the pre- and post-diagnosis physical activity level and can provide actionable recommendations for physical activity after a cancer diagnosis. There is emerging data that maintaining or increasing physical activity from before to after a breast cancer diagnosis can improve survival [2–6] and individual health outcomes following a cancer diagnosis, compared to remaining inactive [7, 8]. Despite the increasing recognition of the importance of change in physical activity, no studies to date investigate how change in physical activity relates to healthcare utilisation of breast cancer survivors, an important metric for both overall quality of survival and healthcare costs.

To address this gap, we conducted a nationwide, longitudinal cohort study using data from multiple national Swedish registers to examine the association of change in physical activity from before to after an incident, nonmetastatic breast cancer diagnosis with the risk of outpatient specialist care and hospitalisations in women with preexisting T2DM.

## Methods

### Data source

Data were ascertained through linkage with Swedish nationwide health and population registers described below. Linkages were made possible through the national personal identification number provided to all Swedish citizens, which is assigned at birth and to immigrants planning to live in Sweden for at least one year [9].

### Study design and population

**Fig. 1:**
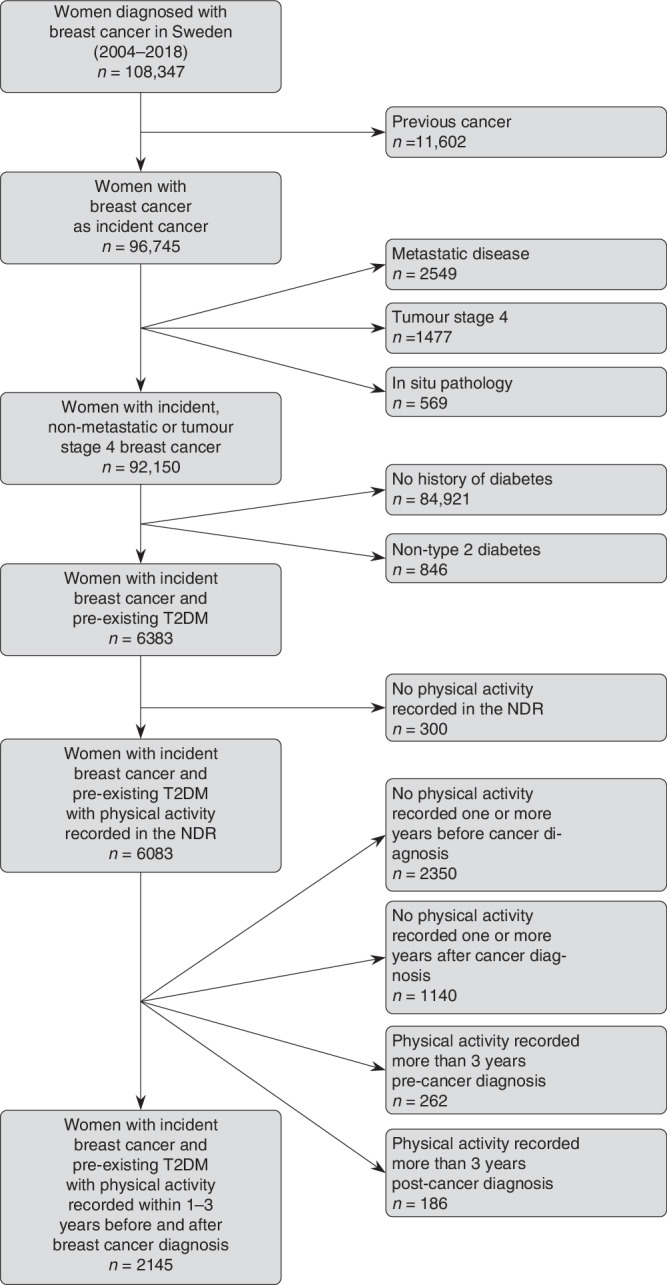
Selection of the study population using the Swedish National Cancer Register and Swedish National Diabetes Register. Abbreviations: T2DM—Type 2 diabetes, NDR—National Diabetes Register.

This population-based cohort study constituted 2145 women aged ≥ 18 years with breast cancer as their incident cancer diagnosed between 2004 and 2018 and preexisting T2DM at least one year prior to cancer diagnosis (identified in the Swedish National Diabetes Register (NDR)) (Fig. 1).

Women were considered to have incident breast cancer if their first ever malignant record in the National Cancer Register, excluding non-melanoma skin cancer, was breast cancer defined according to ICD-10 code “C50”. Cases with pathology consistent with in situ disease (*n* = 569) were excluded. Given the differences in management and characteristics of advanced disease compared to earlier stages, cases with tumor stage 4 disease (*n* = 1477) and metastases (*n* = 2549) at diagnosis were excluded (Fig. 1). Additionally, women were required to have a physical activity measure recorded in the NDR 1–3 years pre- and post-breast cancer diagnosis. Women who did not meet these criteria were excluded (*n* = 3938). As such, all women in the study were diagnosed with T2DM at least one year prior to their breast cancer diagnosis.

### Exposure assessment

In Sweden, physicians or specialised diabetes nurses routinely ask patients with T2DM about their level of physical activity during an annual diabetes-specific visit or visits to address a diabetes-related complication or follow-up.

Physical activity in the NDR is recorded in 5 categories based on the number of times “at least a 30-minute walk or equivalent” was performed per week: “Never,” “<1 time/week,” “1–2 times/week,” “3-4 times/week,” and “Daily.” Coverage for this variable has improved over time and, by 2019, stabilised at 74% in primary care diabetes visits [10]. Previously, a simple self-reported measure of physical activity has been associated with maximum exercise capacity and capillary density, while a very similar question in the SWEDEHEART register on moderate to vigorous intensity physical activity has moderate correlation with accelerometer data (correlation coefficient: 0.3) [11, 12].

We used physical activity recordings at two different time points to determine change in physical activity: before breast cancer diagnosis (pre-diagnosis physical activity) and after breast cancer diagnosis (post-diagnosis physical activity). To reduce the risk of including physical activity levels impacted by cancer symptoms (e.g., fatigue and unintentional weight loss), pre-diagnosis physical activity was defined as the last physical activity record at least 12 months prior to breast cancer diagnosis. Similarly, to avoid including physical activity levels impacted by surgical and/or chemotherapy treatment itself, post-diagnosis physical activity was defined as the first physical activity record at least 12 months after the breast cancer diagnosis (Supplementary Fig. 1). In order to capture physical activity around the time of the breast cancer diagnosis, measurements were limited to no more than 36 months pre- or post- breast cancer diagnosis.

Change from pre- to post-diagnosis physical activity was categorised as: maintained inactive (<3 times/week both before and after cancer diagnosis [reference group]), increased activity (<3 times/week before cancer diagnosis, but ≥3 times/week after diagnosis), decreased activity (≥3 times/week before cancer diagnosis, but <3 times/week after diagnosis) and maintained active (≥3 times/week both before and after cancer diagnosis).

### Outcome assessment

Beginning at the date of each woman’s post-diagnosis physical activity record, outcome data were obtained from the Swedish National Patient Register, a nationwide register with obligatory and complete nationwide reporting of outpatient specialist care and hospitalisations throughout the study period [13, 14]. For each visit, a primary diagnosis in ICD format and up to 30 secondary diagnoses are recorded. Non-specialist outpatient data, i.e., primary care, is not included. A primary diagnosis is listed for over 99% of hospital discharges, with positive predictive values ranging from 85-95% for specific diagnoses that have been validated against medical charts [15].

We had two types of outcomes in our study: count data and time-to-event. For count data, we summed, at the individual level, number of outpatient specialist care visits, total number of days hospitalised, and number of hospitalisations from all records in the Swedish National Patient Register in one of the ICD-10 chapters of interest (Supplementary Table 1) beginning at the date of each woman’s post-diagnosis physical activity record [16]. Groups XV (pregnancy complications), XVI (complications related to fetus and newborn), XVII (congenital malformations), XX (external causes of illness and death), XXI (factors of importance for the state of health), and XXII (codes for special purposes) were excluded due to lack of relevance to the study population.

Our first time-to-event outcome was the first recorded hospitalisation with a main diagnosis in any one of the ICD-10 chapters of interest. Our second time-to-event outcome was the first record in outpatient specialist care or hospitalisation with a main diagnosis in each of the ICD-10 chapters of interest. To provide further descriptive information, we identified primary diagnoses at the three- character ICD-10 category level, for example A46—Erysipelas, that occurred thirty or more times in hospitalisations and fifty or more times in outpatient specialist care.

### Covariate assessment

The covariates were selected based on established risk factors of breast cancer complications in literature and the assumed causal framework shown by a directed acyclic graph (Supplementary Fig. 2). Glycosylated hemoglobin (HbA1c) was considered a possible mediator so was not adjusted for. All models described below were adjusted for possible confounders: smoking status, body mass index (BMI), age at breast cancer diagnosis, tumor stage, node stage, highest education, marital status, endocrine treatment for breast cancer and Charlson Comorbidity Index (CCI).

The National Diabetes Register (NDR) was founded in 1996. In 2019, it was estimated to include 87% of individuals with diabetes in Sweden age 18 years or older [10]. It provided data on smoking (never, former, current), weight (in kgs), and height (in meters). BMI was calculated using the formula weight/height^2^. Data on covariates from the NDR were ascertained from the same record as post-diagnosis physical activity.

The National Cancer Register provided information on the date and age at cancer diagnosis and cancer characteristics, including TNM staging. This register provides nationwide coverage of primary tumor morphology, location, histology, basis of diagnosis, date of diagnosis, and, since 2004, grade for all malignant and certain benign tumors [17, 18]. Reporting to the National Cancer Register has been mandatory by statute since 1958, and completeness is approximately 96% [17]. For those individuals with missing staging information, we extracted stage, where available, from the National Quality Register for Breast Cancer. A prior validation study demonstrated 70-99% agreement for the staging variables in the National Quality Register for Breast Cancer compared with medical records [19].

The Longitudinal Integrated Database for Health Insurance and Labor Market Studies, with data from 1990 onward, provided information on the level of education (categorised as: 9 years or less, 10–12 years, >12 years) and marital status (categorized as: never married, married/registered partner, widow/divorced) from the year prior to the post-diagnosis physical activity measurement.

Given endocrine therapy for breast cancer was likely ongoing at the time of post-cancer diagnosis physical activity measure, we ascertained all prescriptions with the L02 ATC code dispensed to participants within 90 days of the post physical activity measure. The Prescription Drug Register, started in 2005, and contains information on all dispensed prescriptions in Sweden, including injections administered in a clinic [20]. Endocrine treatment at the time of post-cancer diagnosis physical activity measure was coded as present or absent.

An unweighted Charlson Comorbidity Index (CCI) was calculated based on the first three discharge diagnoses for each hospitalisation in the National Patient Register for the ten years before breast cancer diagnosis [21]. Diabetes (uncomplicated and complicated) and breast cancer diagnoses were excluded from the CCI calculation as both diagnoses are inclusion criteria for the study population. CCI was then categorized as 0, 1, and 2 or more comorbidities.

### Statistical analysis

For the count analysis (number of outpatient specialist care visits, number of hospitalisations, days hospitalised) participants were followed from their post-diagnosis physical activity record until migration, death, or end of study period (December 31, 2019).

Incidence rate ratios (IRRs) with 95% confidence intervals (CIs) for the number of outpatient specialist care visits were calculated using multivariable negative binomial models due to over-dispersed but not zero-inflated data. IRR for days hospitalised and number of hospitalisations were estimated using multivariable zero-inflated models. Due to over-dispersed data for days hospitalised, we selected a multivariable, zero-inflated negative binomial model. We used a multivariable, zero-inflated Poisson model for number of hospitalisations.

For the time-to-event analysis, all participants were followed from their post-diagnosis physical activity record until the outcome, death, emigration, or end of the study period (December 31, 2019). Immigration and emigration were ascertained from the Total Population Register, which captures 95% of immigrations and 91% of emigrations within 30 days [22]. The date of death was ascertained from the Cause of Death register, which dates back to 1952 [23].

To estimate hazard ratios (HRs) with 95% confidence intervals for risk of hospitalisation during follow-up, we used a multivariable Cox proportional hazard model adjusted for the minimal sufficient adjustment set with calendar time as underlying timescale. To estimate HRs with 95% confidence intervals for the first ICD-10 chapter-specific record in outpatient specialist care or hospitalisation, we used a series of multivariable Cox proportional hazard models. The hazard ratios from our models represent the weighted average of the hazard over the follow-up period.

Missing data for covariates ranged from 1.2% (education) to 14.8% (BMI) and were imputed using 15 imputations via chained equations using the MICE package in R [24, 25]. The models included the outcome variable, follow-up time, the Nelson Aalen estimator, HbA1c, and the confounder set discussed above.

To explore the risk that those who decreased activity or maintained inactive were more ill than women who maintained or increased activity and, thus, had physical activity measured later after breast cancer diagnosis with the risk of introducing immortal time bias, we calculated the time from pre-diagnosis physical activity measure to cancer diagnosis and from cancer diagnosis to post-diagnosis physical activity. We present this descriptively by change in physical activity level and by participant characteristics.

To assess the robustness of our hazard models to unmeasured confounding, we calculated *E*-values for the point estimate of the hazard ratio and the confidence interval bound closest to one [26]. The *E*-value represents the strength of unmeasured confounding needed to reduce the association between the exposure and outcome to null [26]. Additional sensitivity analysis included restricting the analysis to a postmenopausal age group (55 years and older), and examining ICD-10 chapter-specific outpatient specialist care and hospitalisations separately.

To further scrutinise our results given baseline differences between the women who maintained inactivity and those who changed or maintained activity, we conducted analyses stratified by education (≤9 years versus 10+ years), CCI (0 versus 1+), BMI (18-25 versus 25+), Tumor stage (tumor not found or <2 cm versus 2 cm or larger), node stage (none positive versus 1 or more positive), and smoking status (never smoker versus former or current smoker) for the risk of experiencing a hospitalisation during follow up and risk of first occurrence of outpatient specialist care or hospitalisation for frequently occurring ICD-10 chapters.

All analyses were run on R version 4.2.1 [27].

## Results

We followed 2145 women (average age 70.4 years) with an incident breast cancer and preexisting T2DM (Fig. 1). Women who maintained inactive tended to have more comorbidities, lower education, higher HbA1c, and larger tumors at diagnosis than other groups (Table 1). They also tended to be current smokers, widows, and slightly older than the other groups. Women who maintained active, increased, or decreased activity were similar in education level, marital status, smoking status, tumor stage, node stage, BMI, and HbA1c.

**Table 1:** Characteristics of women with incident, nonmetastatic breast cancer diagnosed from 2004 to 2018 and preexisting T2DM in Sweden by change in physical activity status.

	Maintained inactive	Increased activity	Decreased activity	Maintained active
	*n* = 785	*n* = 294	*n* = 423	*n* = 643
Age at cancer diagnosis (years)				
Mean [SD]	72.0 [9.9]	68.5 [9.1]	70.3 [9.7]	69.3 [8.7]
Tumor stage, *n* (%)				
primary tumor not found	68 (9)	26 (9)	40 (10)	68 (10)
<2 cm	398 (51)	161 (55)	220 (52)	360 (56)
2–5 cm	267 (34)	91 (31)	146 (34)	188 (29)
>5 cm	40 (5)	15 (5)	12 (3)	19 (3)
Node stage, *n* (%)				
absent	647 (82)	235 (80)	358 (85)	529 (82)
1–3 positive nodes	110 (14)	52 (18)	56 (13)	97 (15)
>3 positive nodes	5 (0.6)	4 (1)	5 (1)	4 (0.6)
Endocrine treatment at time of post-cancer diagnosis physical activity measure, *n* (%)				
Present	587 (75)	229 (78)	319 (75)	487 (76)
Absent	198 (25)	65 (22)	105 (25)	156 (24)
Highest education Level, *n* (%)				
9 years or less	347 (44)	94 (32)	161 (38)	229 (35)
10–12 years	308 (39)	134 (45)	176 (42)	287 (45)
13+ years	116 (15)	65 (22)	80 (19)	123 (19)
Marital status, *n* (%)				
Never married	86 (11)	35 (12)	31 (7)	64 (10)
Married/ registered partner	339 (43)	137 (47)	217 (51)	347 (54)
Widow/divorced	360 (46)	121 (41)	174 (41)	231 (36)
Smoking status, *n* (%)				
Never	489 (62)	168 (57)	266 (63)	413(64)
Former	177 (23)	93 (32)	97 (23)	179(28)
Current	99 (13)	22 (8)	48 (11)	46 (7)
CCI, *n* (%)				
0	408 (52)	203 (69)	279 (66)	464 (72)
1	249 (32)	70 (24)	105 (25)	151 (24)
2+	128 (16)	21 (7)	39 (9)	28 (4)
HbA1c (mmol/mol)				
Mean [SD]	57.6 [15]	54.5 [11]	55.9 [13]	53.5 [12]
BMI (kg/m^2^)				
Mean [SD]	31.3 [5.7]	29.6 [4.9]	29.8 [4.8]	28.2 [4.3]

*T2DM* type 2 diabetes, *SD* standard deviation, *CCI* unweighted Charlson Comorbidity index, *HbA1c* glycosylated hemoglobin, *BMI* body mass index.

The mean time from the pre-diagnosis physical activity measure to breast cancer diagnosis was 1.6 years (SD 0.5), and from breast cancer diagnosis to post-diagnosis physical activity measure was 1.6 years (SD 0.5). These values were similar when stratified by change in physical activity level and other participant characteristics (Supplementary Table 2).

The median follow-up time from the post-diagnosis physical activity measure to the first outpatient specialist care visit was 0.4 years (IQR 0.1–1.0 years) with a total person-time of 1738 years. The median follow-up time from the post-diagnosis physical activity measure to the first record of hospitalisation was 1.7 years (IQR 0.7–3.6 years) with a total person time of 5164 years. For the ICD-10 chapter-specific outcomes, follow-up time ranged from a median of 1.68 years (IQR 0.63–3.52) in the nervous, eye, and ear chapters to a median of 3.2 years (IQR 1.53 –5.64) in the blood disorders chapter. Total person time ranged from 5129 person years to 8066 person years.

### Frequency of outpatient specialist care visits, hospitalisations, and days hospitalised

In total, 20091 outpatient specialist care visits occurred and 3528 hospitalisations resulted in 24662 days hospitalised. Those who maintained active or increased activity experienced a lower rate of outpatient specialist care visits compared to women who maintained inactive (IRR 0.74 (0.66–0.83) and IRR 0.84 (0.74–0.97), respectively), while women who decreased activity had similar rates of outpatient specialist care visits per year as women who maintained inactive. In comparison to women who maintained inactive, those who maintained active or increased activity experienced 40% lower rate of days hospitalised (IRR 0.57 (0.45–0.73) and 0.61 (0.45–0.83), respectively) and 30% lower rate of hospitalisations (IRR 0.71 (0.64–0.79) and IRR 0.72 (0.63–0.83), respectively) (Table 2). To a lesser extent than those who maintained active or increased activity, those women who decreased activity also experienced a lower rate of days hospitalised and hospitalisations than those who maintained inactive (Table 2).

**Table 2:** Number of outpatient specialist care visits, days hospitalised, and number of hospitalisations per year of follow-up in ICD chapters of interest (i.e. ICD-10 chapters: 1–14, 18, 19) for 2145 women with incident, nonmetastatic breast cancer diagnosed from 2004 to 2018 and preexisting T2DM by a change in physical activity status from before to after a cancer diagnosis.

	Physical Activity status	Maintained inactive	Increased activity	Decreased activity	Maintained active
		(*n* = 785)	(*n* = 294)	(*n* = 423)	(*n* = 643)
Specialist outpatient care visits	Total outpatient specialist care visits (*n*)	7800	2558	4654	5079
	Median outpatient specialist care visits per year of follow-up (IQR)	1.96 (0.88–4.19)	1.59 (0.49–3.49)	1.88 (0.79–3.78)	1.38 (0.47–2.74)
	IRR^c^ (95% CI)	1.00 (reference)	0.84 (0.74–0.97)	0.96 (0.86–1.08)	0.74 (0.66–0.83)
Days hospitalised	Total days hospitalised (*n*)	12191	2298	5198	4975
	Median days hospitalised per year of follow-up (IQR)	1.00 (0–6.15)	0 (0–1.62)	0.48 (0–3.64)	0 (0–1.25)
	IRR^a^ (95% CI)	1.00 (reference)	0.61 (0.45-0.83)	0.78 (0.60-0.97)	0.57 (0.45–0.73)
Hospitalisations	Total hospitalisations (*n*)	1630	365	781	752
	Median number of hospitalisations per year of follow-up (IQR)	0.27 (0–0.91)	0 (0–0.39)	0.20 (0–0.60)	0 (0–0.36)
	IRR^b^ (95% CI)	1.00 (reference)	0.72 (0.63–0.83)	0.87 (0.79–0.96)	0.71 (0.64–0.79)

*T2DM* type 2 diabetes, *IQR* inter quartile range, *IRR* incidence rate ratio, *BMI* body mass index, *CCI* unweighted Charlson Comorbidity index, *CI* confidence interval.

^a^Multivariable zero-inflated negative binomial model adjusted for smoking status, BMI, age at breast cancer diagnosis, tumor stage, node stage, highest education, marital status, endocrine treatment and CCI.

^b^Multivariable zero-inflated Poisson model adjusted for smoking status, BMI, age at breast cancer diagnosis, tumor stage, node stage, highest education, marital status, endocrine treatment and CCI.

^c^Multivariable negative binomial model adjusted for smoking status, BMI, age at breast cancer diagnosis, tumor stage, node stage, highest education, marital status, endocrine treatment and CCI.

ICD-10 Chapter 18, which describes general symptoms not classified elsewhere (Supplementary Table 1), was the most common chapter for the main diagnosis for first-occurrence outpatient specialist care or hospitalisation overall (*n* = 980) and for outpatient specialist care records alone (*n* = 949) (Supplementary Table 3). Circulatory diseases were the most common diagnosis for hospitalisations (*n* = 381). The specific diagnoses at the category level with 30 or more first-occurrence diagnoses in hospitalisations or 50 or more first-occurrence diagnoses in outpatient specialist care are listed in Supplementary Table 4.

### Rate of ever experiencing a hospitalisation during follow-up and rates of outpatient specialist care visit or hospitalisation visits by ICD-10 chapter

**Fig. 2:**
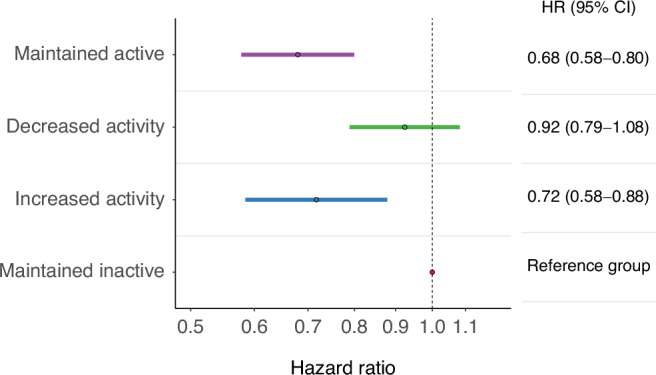
Rate of ever experiencing a hospitalisation during follow-up by change in physical activity level. Data are hazard ratios (HR) of ever experiencing a hospitalization by change in physical activity level in 2145 women with incident, nonmetastatic breast cancer diagnosed between 2004 and 2018 and preexisting type 2 diabetes in Sweden. The Cox proportional hazard model was adjusted for smoking status, body mass index, age at breast cancer diagnosis, tumor stage, node stage, highest education, marital status, endocrine treatment, and unweighted Charlson Comorbidity Index. Abbreviations: CI—Confidence Interval.

**Fig. 3:**
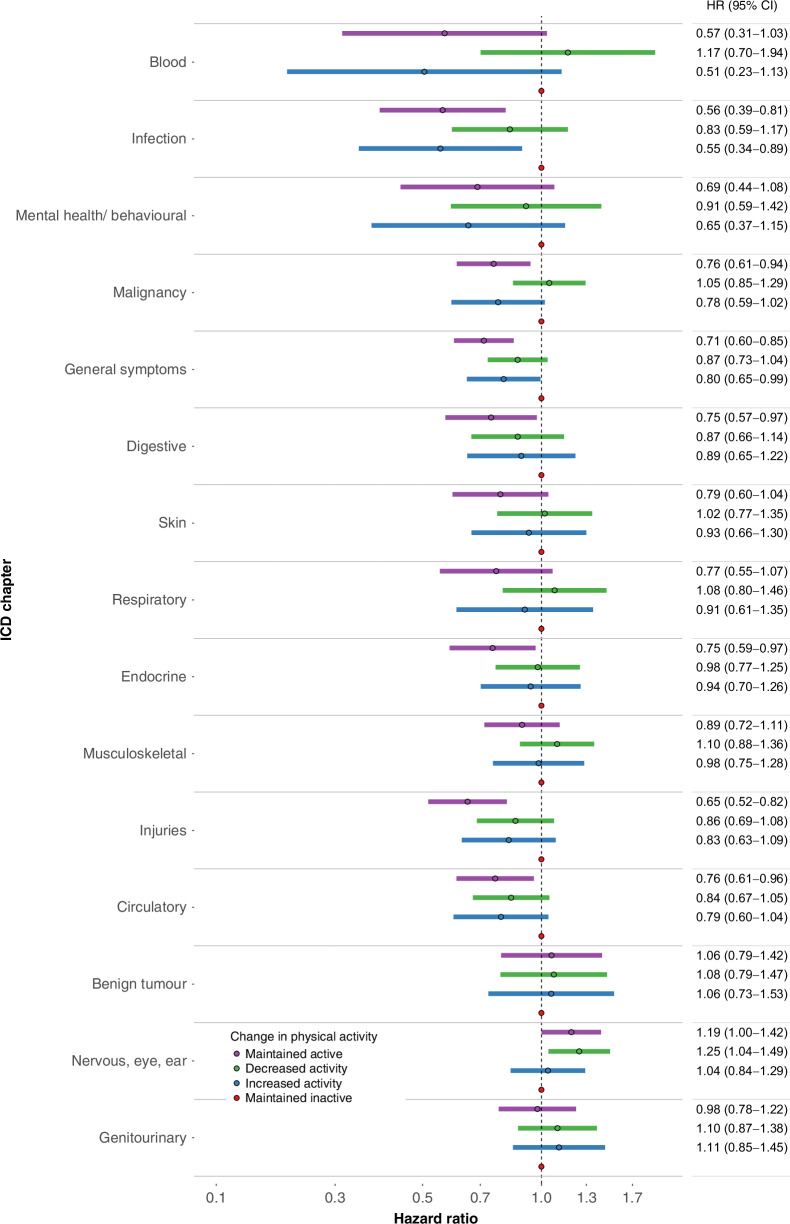
ICD-10 chapter specific rates of outpatient specialist care visit or hospitalisation by change in physical activity level. Data are hazard ratios (HR) and 95% confidence interval (95% CI) of first occurance outpatient specialist care or hospitalisation by International Classification of Disease (ICD)-10 chapter among 2145 women with incident, nonmetastatic breast cancer diagnosed between 2004 and 2018 and pre-existing type 2 diabetes in Sweden. Cox proportional hazard models were adjusted for smoking status, body mass index, age at breast cancer diagnosis, tumor stage, node stage, highest education, marital status, endocrine treatment, and unweighted Charlson Comorbidity Index. Abbreviations: CI—Confidence Interval.

The majority of associations between change in physical activity and diagnoses in outpatient specialist care and hospitalisations could be group into two main patterns. Firstly, women who maintained active or increased activity, in comparison to those women who maintained inactive, experienced an ~30% lower rate of hospitalisation during follow up (Fig. 2, HR 0.68 [0.58–0.80] & 0.72 [0.58–0.88]). A similar magnitude of effect was seen for incident outpatient specialist care visit or hospitalisation within the ICD-10 chapters of blood, infection, mental health and behavioural, malignancy, and general symptoms (Fig. 3).

Secondly, women who maintained active, but not those who increased or decreased activity, experienced lower rates of care within a number of ICD chapters compared to those who maintained inactive. This was the pattern for digestive diseases (HR 0.75 [0.57–0.97]) with a similar magnitude of effect for skin, respiratory, endocrine diseases, musculoskeletal, and injuries ICD-10 chapters (Fig. 3).

The results for the remaining four ICD-10 chapters did not fit either of the two main patterns. Instead, women who maintained active, increased, or decreased activity experienced lower rate for diseases of the circulatory system compared to those who maintained inactive while all three groups experienced no difference for benign tumors and genitourinary diagnoses (Fig. 3). Those who maintained active and decreased activity experienced more care related to nervous, eye and ear diagnoses (Fig. 3) compared to those who maintained inactive.

**Fig. 4:**
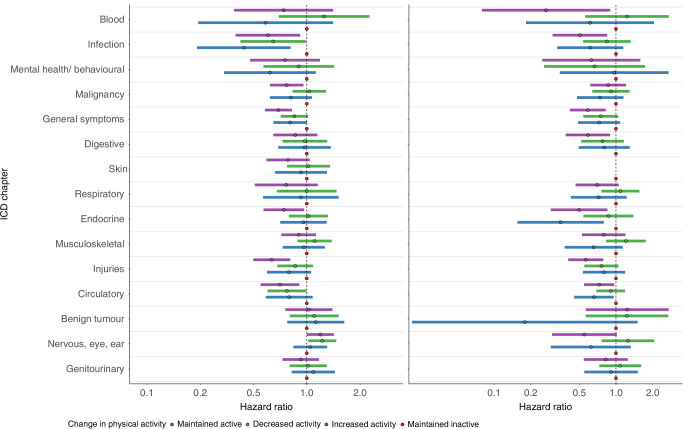
ICD-10 chapter specific rates of first occurrence outpatient specialist care (left panel) and hospitalisations (right panel) by change in physical activity level. Data are hazard ratios (HR) and 95% confidence interval (95% CI) of first occurrence outpatient specialist care (left) and hospitalizations (right) by International Classification of Disease-10 (ICD) chapter among 2145 women with incident, nonmetastatic breast cancer diagnosed between 2004 and 2018 and pre-existing type 2 diabetes in Sweden. Cox proportional hazard models were adjusted for smoking status, body mass index, age at breast cancer diagnosis, tumor stage, node stage, highest education, marital status, endocrine treatment, and unweighted Charlson Comorbidity Index. Skin diagnoses for hospitalizations were omitted due to few cases (*n* = 21).

The same pattern of hazard ratios was observed in sensitivity analyses of a postmenopausal age group (women 55 years old or older at time of breast cancer diagnosis) as was seen in the main analyses (Supplementary Figs. 3 and 4). The results also generally followed the main analyses when outpatient specialist and hospitalisations were examined separately (Fig. 4). Although, for hospitalisations, women who increased activity compared to those who maintained inactive experienced a lower hazard for care related to ICD-10 chapters of respiratory, endocrine, musculoskeletal, benign tumors, and nervous, eye and ear diseases, where this was not observed in the outpatient specialist care and main analyses (Fig. 4). Results for analyses stratified by participant characteristics (BMI, CCI, education, node and tumor stage, smoking status) were largely in line with the main results (Supplementary Fig. 5).

*E*-values for the hazard ratios from the main analysis ranged from 1.13 (skin) to 3.37 (blood disorders) and for the bound of the 95% CI closest to 1: 1 to 1.77 (Supplementary Table 5).

## Discussion

In this register-based cohort study, women with incident breast cancer and preexisting T2DM who reported being physically active at least three days a week both before and after a breast cancer diagnosis experienced less usage of outpatient specialist care and fewer hospitalisations compared to women who were consistently inactive. Notably, an increase from physical activity fewer than three days per week before diagnosis to three or more days per week after diagnosis also resulted in lower outpatient specialist care usage and fewer hospitalisations across a range of ICD-10 chapters. We also noticed several patterns in the associations between change in physical activity and ICD-10 chapter diagnoses in outpatient specialist care and for hospitalisations. For ICD chapters where women who increased activity or maintained active experienced lower rates of care than women who maintained inactive, diagnoses in these chapters are possibly more responsive to short-term physical activity trends. In contrast, for the chapters where only women in the maintains active group experienced lower rates of care compared to women who maintained inactive, the effects of physical activity may accrue over a longer duration of time. Overall, however, regardless of physical activity level, most women in our study experienced few outpatient specialist and hospitalisations per year of follow-up.

Our study adds to the limited body of literature examining the association between changes in physical activity and breast cancer outcomes. Our findings are consistent with recent population based cohort studies of middle-aged women with breast cancer that demonstrated better overall survival for women who increased or maintained a high level of physical activity [4, 5]. Our study is consistent with recent cohort and pragmatic follow-up studies focused on post cancer diagnosis physical activity. These studies reported a lower risk of individual late effects, such as cardiovascular disease, stroke, and cognitive function, in physically active cancer survivors [28–30]. Our study provides novel insights for a broader range of diagnoses than previously described and enabled us to disentangle post-breast cancer diagnosis physical activity from pre-breast cancer diagnosis physical activity. Studies focused on pre-diagnosis physical activity alone are limited in their ability to lead to actionable interventions for women newly diagnosed with breast cancer. Studies focused on post-diagnosis physical activity are challenging to interpret as they inherently lack information on the pre-diagnosis activity, making it unclear if the benefit of physical activity is due to the lasting effects of the pre-diagnosis activity level and underlying fitness or if it represents an actual impact of the post-diagnosis activity level.

Randomised control studies have shown physical activity improves quality of life and physical function in cancer patients [31]. Cancer patients who volunteer for a randomised control trial, however, are a select group, limiting generalisability. Observational studies may better reflect the general cancer patient population. A growing body of observational literature suggests that maintaining or increasing activity to a modest amount of physical activity can benefit health [3–6]. The Nurses’ Health Study recently found that increasing physical activity by the equivalent of 1–3 h per week of walking at 2.5 mph was associated with a decrease in breast-cancer-specific mortality [4]. In stage three colon cancer, patients who engaged in at least 1.5 h per week of light to moderate physical activity at less than 6 metabolic equivalents had a 21.4% absolute higher rate of three-year disease-free survival than patients who did not engage in physical activity [32]. Our results show that maintaining or increasing walking or equivalent to as little as 30 min, three days or more per week may be beneficial for women with breast cancer and T2DM. This definition of active, 30 min of walking three or more days per week, is in line with Swedish recommendations, which indicate that individuals with T2DM should be physically active 3–7 times per week [33] and with an international consensus statement recommending of 30 min of activity, 3–4 days per week for cancer survivors [34]. The threshold of 3 times/week for the definition of active has also been used previously when physical activity has been examined in the NDR [35–37].

Qualitative research indicates that cancer survivors desire more specific recommendations from their oncology team on physical activity [38, 39] and are interested in home-based physical activity, such as walking [39, 40]. Our study, alongside recent survival research, adds valuable morbidity data to the growing body of evidence supporting moderate physical activity as a feasible and beneficial intervention for breast cancer survivors, especially those with T2DM, which can be used to inform clinical recommendations desired by cancer patients.

A major strength to this study is the population-based approach using high-quality national register data. This allows the results of our study to have better generalizability than, for example, randomised trials, wherein participation tends to be skewed towards individuals already inclined towards such activities. Physical activity in our study was measured before the outcome of interest occurred, reducing the potential for recall bias. Additionally, based on our study design, we could provide a broad description of survivor morbidity. This study’s physical activity measurement (walking 30 min per day or equivalent) does not necessitate adherence to structured programmes or supervision to be used as an intervention in the future, enhancing its utility in real-world settings. Previous attempts to examine change in physical activity from before to after a cancer diagnosis have been based on cohort studies where data are collected at a predetermined interval meaning there could be long intervals between physical activity assessments [2, 3]. This makes it difficult to assess the physical activity level shortly after a breast cancer diagnosis and lengthens the time a woman must survive to be included in the study. The high-resolution physical activity data recorded in the National Diabetes Register means that this limitation is substantially less of a problem in our study than with other data sources.

Our study has limitations. By comparing the change in physical activity level from one year before to one year after a breast cancer diagnosis, our study is limited to those women who survived at least one year after cancer diagnosis. Women diagnosed with breast cancer during our study period, however, had an expected one-year survival of over 97% [41]. Additionally, there was the possibility for systematic differences in the time from the pre-diagnosis physical activity measure to cancer diagnosis and from cancer diagnoses to the post-diagnosis physical activity across groups in our study. However, we found the time between physical activity measures and cancer diagnosis were similar across all groups which gave us confidence that such mechanisms did not drive the results. The possibility of reverse causality where individuals with a higher likelihood of hospitalisation may engage in lower amounts of physical activity due to functional limitations from illness or frailty cannot be ruled out. We sought to minimise the potential for reverse causality by beginning post-breast cancer diagnosis physical activity measurement a minimum of one year after diagnosis. Additionally, women who increased and decreased their physical activity were comparable in terms tumor characteristics, Charlson comorbidity index, BMI, smoking status and time between physical activity measures and cancer diagnosis, making it unlikely that reverse causality significantly influences our findings. Finally, the physical activity measure was an oral, self-reported measure that, while an inexpensive and rapid assessment, may not be as accurate as a physical measurement such as an accelerometer. However, this method for measuring physical activity is advantageous as it is highly relevant to the general population, reflecting women’s perceived physical activity level. Further, a validation study which also ranked individuals’ self-reported physical activity within groups found general agreement between this type of physical activity measure and physiologic measurements [12].

Unmeasured confounding is always a concern of observational studies. *E*-values suggest unmeasured confounding, such as via tumor hormone status or breast cancer treatment, would require a modestly strong relation to both changes in physical activity and healthcare utilisation to negate our findings (Supplementary Table 5). Participation in our study was also contingent upon having both T2DM and breast cancer thus selection bias via a collider bias is possible [42]. However, given that our study included many possible confounders due to extensive linkage between national registers, we do not think residual confounding or collider bias is a likely explanation for our results. Additionally, analyses stratified by a range of covariates for multiple outcomes demonstrated a similar pattern to the main findings, supporting our results’ stability and that the results could be generalised to subgroups.

Overall, women with incident breast cancer and preexisting T2DM who walk 30 min per day, most days, both before and after an incident breast cancer diagnosis, or who increase their physical activity after their breast cancer diagnosis experienced fewer hospitalisations and outpatient specialist care visits compared to inactive women. Moderate, physical activity, such as walking, should be encouraged throughout the cancer care continuum, independent of a woman’s physical activity level prior to a breast cancer diagnosis. Future studies evaluating physical activity trajectories over a longer period of time are needed to understand the relationship between nuances in long-term physical activity patterns and breast cancer outcomes.

## Supplementary information

Supplementary Tables and Figures

STROBE checklist

## Data Availability

The data that support the findings of this study are available from Statistics Sweden and the National Board of Health and Welfare but restrictions apply to the availability of these data, which were used under license for the current study, and so are not publicly available. Researchers can, after ethical approval, apply for the data from Statistics Sweden and the Swedish National Board of Health and Welfare.
