# Supplementary material for: Association of change in physical activity with use of outpatient specialist care and hospitalisations among breast cancer survivors with type 2 diabetes in Sweden
Source: Br J Cancer. 2025 Jul 4;133(5):655–64. doi: 10.1038/s41416-025-03099-x (PMC12405502; doi:10.1038/s41416-025-03099-x)
Supplement: Supplementary file 1 — Supplementary Tables and Figures [file 41416_2025_3099_MOESM1_ESM.docx]

Supplementary Tables and Figures accompanying the article:

*Association of change in physical activity with use of outpatient specialist care and hospitalizations among breast cancer survivors with type 2 diabetes in Sweden*

Genevieve Allen^1^, Emerald G Heiland^1^, Stanley Teleka^2^, Ingrid Glimelius^3^, Karl Michaëlsson^1^, Liisa Byberg^1^, Hannah L Brooke*^1^

^1^ Medical Epidemiology, Department of Surgical Sciences, Uppsala University

^2^Department of Clinical Sciences, Malmö, Lund University

^3^ Department of Immunology, Genetics and Pathology, Unit of Cancer Precision Medicine, Uppsala University

*Corresponding Author ORCID: 0000-0002-7085-1551

*Corresponding Author email: [hannah.brooke@uu.se](mailto:hannah.brooke@uu.se)

Contents

[**Supplementary Table 1.** 2](#_Toc196479926)

[**Supplementary Table 2** 3](#_Toc196479927)

[**Supplementary Table 3.** 4](#_Toc196479928)

[**Supplementary Table 4.** 5](#_Toc196479929)

[**Supplementary Figure 1.** 8](#_Toc196479930)

[**Supplementary Figure 3.** 10](#_Toc196479931)

[**Supplementary Figure 4.** 11](#_Toc196479932)

[**Supplementary Figure 5** 13](#_Toc196479933)

| Supplementary Table 1. Diagnosis groups by International Classification of Disease (ICD)-10 chapter. | | |  |
| --- | --- | --- | --- |
| ICD Chapter Number | Diagnosis group | ICD-10 code | |
| I | Infection | A00-B99 | |
| II | Malignancy | C00-C99 | |
| III | Benign Tumor | D0-D48 | |
| III | Blood disorders | D50-D89 | |
| IV | Endocrine | E00-E90 | |
| V | Mental health and behavioral | F00-F99 | |
| VI, VII, VIII | Nervous, eye, ear | G00-G99 + H00-H95 | |
| IX | Circulatory | I00-I99 | |
| X | Respiratory | J00-J99 | |
| XI | Digestive | K00-K93 | |
| XII | Skin | L00-L99 | |
| XIII | Musculoskeletal | M00-M99 | |
| XIV | Genitourinary | N00-N99 | |
| XVIII | General symptoms | R00-R99 | |
| XIX | Injuries | S00-T98 | |

| Supplementary Table 2. Mean time in years from pre-diagnosis physical activity measure to cancer diagnosis and from cancer diagnoses to post-diagnosis physical activity measure stratified by change in physical activity and other participant characteristics among 2145 women with incident, nonmetastatic breast cancer diagnosed between 2004 and 2018 and preexisting type 2 diabetes. | | |
| --- | --- | --- |
|  | Time from pre-diagnosis physical activity measure to cancer diagnosis in years  Mean (SD) | Time from cancer diagnoses to post-diagnosis physical activity measure in years  Mean (SD) |
| Physical activity status |  |  |
| Maintained inactive | 1.59 (0.47) | 1.59 (0.46) |
| Increased activity | 1.54 (0.45) | 1.55 (0.44) |
| Decreased activity | 1.66 (0.49) | 1.58 (0.45) |
| Maintained active | 1.57 (0.45) | 1.58 (0.45) |
| Tumor stage |  |  |
| primary tumor not found | 1.62 (0.50) | 1.64 (0.47) |
| <2 cm | 1.58 (0.45) | 1.58 (0.45) |
| 2-5 cm | 1.60 (0.49) | 1.56 (0.45) |
| >5 cm | 1.59 (0.48) | 1.59 (0.46) |
| Node stage |  |  |
| absent | 1.59 (0.47) | 1.58 (0.45) |
| 1-3 positive nodes | 1.59 (0.48) | 1.57 (0.45) |
| >3 positive nodes | 1.92 (0.51) | 1.56 (0.31) |
| CCI |  |  |
| 0 | 1.60 (0.47) | 1.58 (0.44) |
| 1 | 1.58 (0.47) | 1.57 (0.45) |
| 2+ | 1.59 (0.46) | 1.65 (0.51) |
| Education |  |  |
| ≤ 9 years | 1.61 (9.49) | 1.58 (0.45) |
| 10-12 years | 1.58 (0.46) | 1.58 (0.45) |
| 13+ years | 1.57 (0.45) | 1.56 (0.44) |
| Smoking |  |  |
| Never smoker | 1.60 (0.51) | 1.57 (0.45) |
| Former smoker | 1.55 (0.44) | 1.60 (0.46) |
| Current smoker | 1.57 (0.47) | 1.60 (0.43) |

Abbreviations: CCI – unweighted Charlson Comorbidity Index; SD – standard deviation

| Supplementary Table 3. Frequency of International Classification of Disease-10 chapter occurrence by healthcare encounter type among 2145 women with incident, nonmetastatic breast cancer diagnosed between 2004 and 2018 and preexisting type 2 diabetes. | | | | | | |  |
| --- | --- | --- | --- | --- | --- | --- | --- |
| Diagnosis group | | First outpatient specialist care or hospitalization (n) | First hospitalization alone  (n) | | | First outpatient specialist care alone  (n) |  |
| Infection | | 241 | 130 | | | 168 |  |
| Malignancy | | 644 | 245 | | | 625 |  |
| Benign tumor | | 324 | 47 | | | 308 |  |
| Blood disorders | | 97 | 39 | | | 78 |  |
| Endocrine | | 497 | 113 | | | 457 |  |
| Mental health/ behavioral | | 149 | 40 | | | 139 |  |
| Nervous, eye, ear | | 956 | 98 | | | 933 |  |
| Circulatory | | 613 | 381 | | | 483 |  |
| Respiratory | | 298 | 197 | | | 190 |  |
| Digestive | | 418 | 179 | | | 357 |  |
| Skin | | 382 | 21 | | | 376 |  |
| Musculoskeletal | | 619 | 182 | | | 591 |  |
| Genitourinary | | 584 | 178 | | | 524 |  |
| Injuries | | 584 | 281 | | | 539 |  |
| General symptoms | | 980 | 273 | | | 949 |  |
| Supplementary Table 4. Main diagnoses at the category level with 50 or more first occurrences in outpatient specialist care (left) and 30 or more first occurrence diagnoses from hospitalizations (right) among 2145 women with incident, nonmetastatic breast cancer diagnosed between 2004 and 2018 and preexisting type 2 diabetes. | | | | | | | |
| Outpatient specialist care diagnoses | | | | **Hospitalization diagnoses** | | | |
| A46 | Erysipelas | | | A46 | Erysipelas | | |
| C50 | Breast cancer | | | C50 | Breast cancer | | |
| E11 | Type 2 diabetes | | | G45 | Transient ischemic attack and related syndromes | | |
| H25 | Age-related cataract | | | I26 | Pulmonary embolism | | |
| I50 | Heart failure | | | I50 | Heart failure | | |
| L57 | Skin changes by chronic exposure to non-ionizing radiation | | | J15 | Bacterial pneumonia, not otherwise classified | | |
| M17 | Knee osteoarthritis | | | J18 | Bacterial pneumonia, organism unspecified | | |
| R06 | Abnormal breathing | | | M17 | Knee osteoarthritis | | |
| R07 | Pain in throat and chest | | | N10 | Acute pyelonephritis | | |
| R10 | Pain in abdomen and pelvis | | | N39 | Other disorders of urinary system | | |
| R42 | Dizziness and vertigo | | | R06 | Abnormal breathing | | |
| S42 | Fracture of shoulder and upper arm | | | R07 | Pain in throat and chest | | |
|  |  | | | R42 | Dizziness and vertigo | | |
|  |  | | | S42 | Fracture of shoulder and upper arm | | |

**Supplementary Table 5. E-values for hazard ratio and confidence interval (CI) closest to one of first occurrences of outpatient specialist care or hospitalization by International Classification of Disease-10 (ICD) chapter among 2145 women with incident, nonmetastatic breast cancer diagnosed between 2004 and 2018 and preexisting type 2 diabetes.**

| **ICD group** | **Change in physical activity group** | **E-value for the hazard ratio** | **E-value for the confidence interval** |
| --- | --- | --- | --- |
| Infection | Maintained Active | 2.96 | 1.77 |
| Infection | Increased Activity | 3.00 | 1.49 |
| Infection | Decreased Activity | 1.69 | 1.00 |
| Malignancy | Maintained Active | 1.72 | 1.26 |
| Malignancy | Increased Activity | 1.67 | 1.00 |
| Malignancy | Decreased Activity | 1.22 | 1.00 |
| Benign Tumor | Maintained Active | 1.25 | 1.00 |
| Benign Tumor | Increased Activity | 1.31 | 1.00 |
| Benign Tumor | Decreased Activity | 1.28 | 1.00 |
| Blood | Maintained Active | 2.91 | 1.00 |
| Blood | Increased Activity | 3.37 | 1.00 |
| Blood | Decreased Activity | 1.61 | 1.00 |
| Endocrine | Maintained Active | 1.73 | 1.18 |
| Endocrine | Increased Activity | 1.26 | 1.00 |
| Endocrine | Decreased Activity | 1.14 | 1.00 |
| Mental health/ behavioral | Maintained Active | 2.26 | 1.00 |
| Mental health/ behavioral | Increased Activity | 2.43 | 1.00 |
| Mental health/ behavioral | Decreased Activity | 1.41 | 1.00 |
| Nervous, eye, ear | Maintained Active | 1.51 | 1.03 |
| Nervous, eye, ear | Increased Activity | 1.19 | 1.00 |
| Nervous, eye, ear | Decreased Activity | 1.60 | 1.20 |
| Circulatory | Maintained Active | 1.70 | 1.21 |
| Circulatory | Increased Activity | 1.63 | 1.00 |
| Circulatory | Decreased Activity | 1.51 | 1.00 |
| Respiratory | Maintained Active | 1.93 | 1.00 |
| Respiratory | Increased Activity | 1.44 | 1.00 |
| Respiratory | Decreased Activity | 1.30 | 1.00 |
| Digestive | Maintained Active | 1.75 | 1.16 |
| Digestive | Increased Activity | 1.39 | 1.00 |
| Digestive | Decreased Activity | 1.43 | 1.00 |
| Skin | Maintained Active | 1.64 | 1.00 |
| Skin | Increased Activity | 1.29 | 1.00 |
| Skin | Decreased Activity | 1.13 | 1.00 |
| Musculoskeletal | Maintained Active | 1.38 | 1.00 |
| Musculoskeletal | Increased Activity | 1.12 | 1.00 |
| Musculoskeletal | Decreased Activity | 1.33 | 1.00 |
| Genitourinary | Maintained Active | 1.14 | 1.00 |
| Genitourinary | Increased Activity | 1.35 | 1.00 |
| Genitourinary | Decreased Activity | 1.34 | 1.00 |
| Injuries | Maintained Active | 2.03 | 1.56 |
| Injuries | Increased Activity | 1.54 | 1.00 |
| Injuries | Decreased Activity | 1.46 | 1.00 |
| General symptoms | Maintained Active | 1.84 | 1.48 |
| General symptoms | Increased Activity | 1.60 | 1.07 |
| General symptoms | Decreased Activity | 1.43 | 1.00 |
| Ever hospitalized | Maintained Active | 1.94 | 1.61 |
| Ever hospitalized | Increased Activity | 1.83 | 1.41 |
| Ever hospitalized | Decreased Activity | 1.30 | 1.00 |


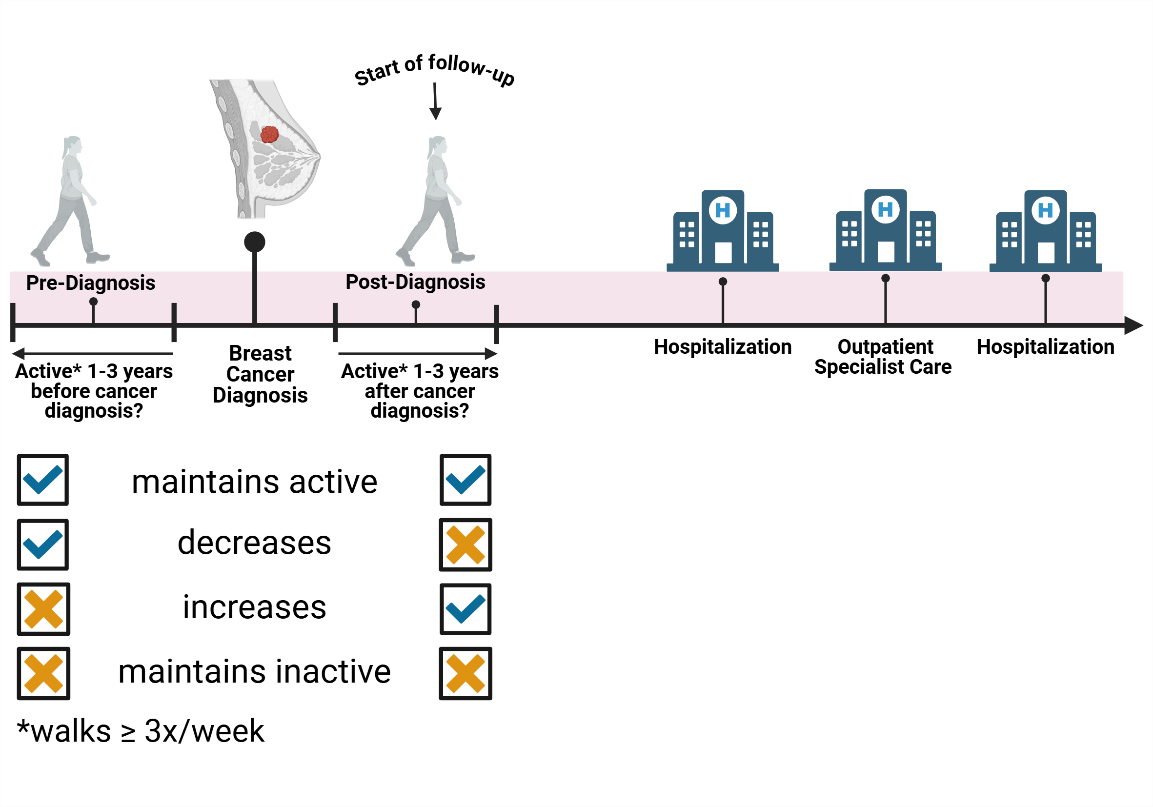


**Supplementary Figure 1.** Study design showing timeline of change in physical activity

assessment in relation to breast cancer diagnosis and outcome ascertainment. Created in BioRender. Brooke, H. (2025) <https://BioRender.com/o58y251>


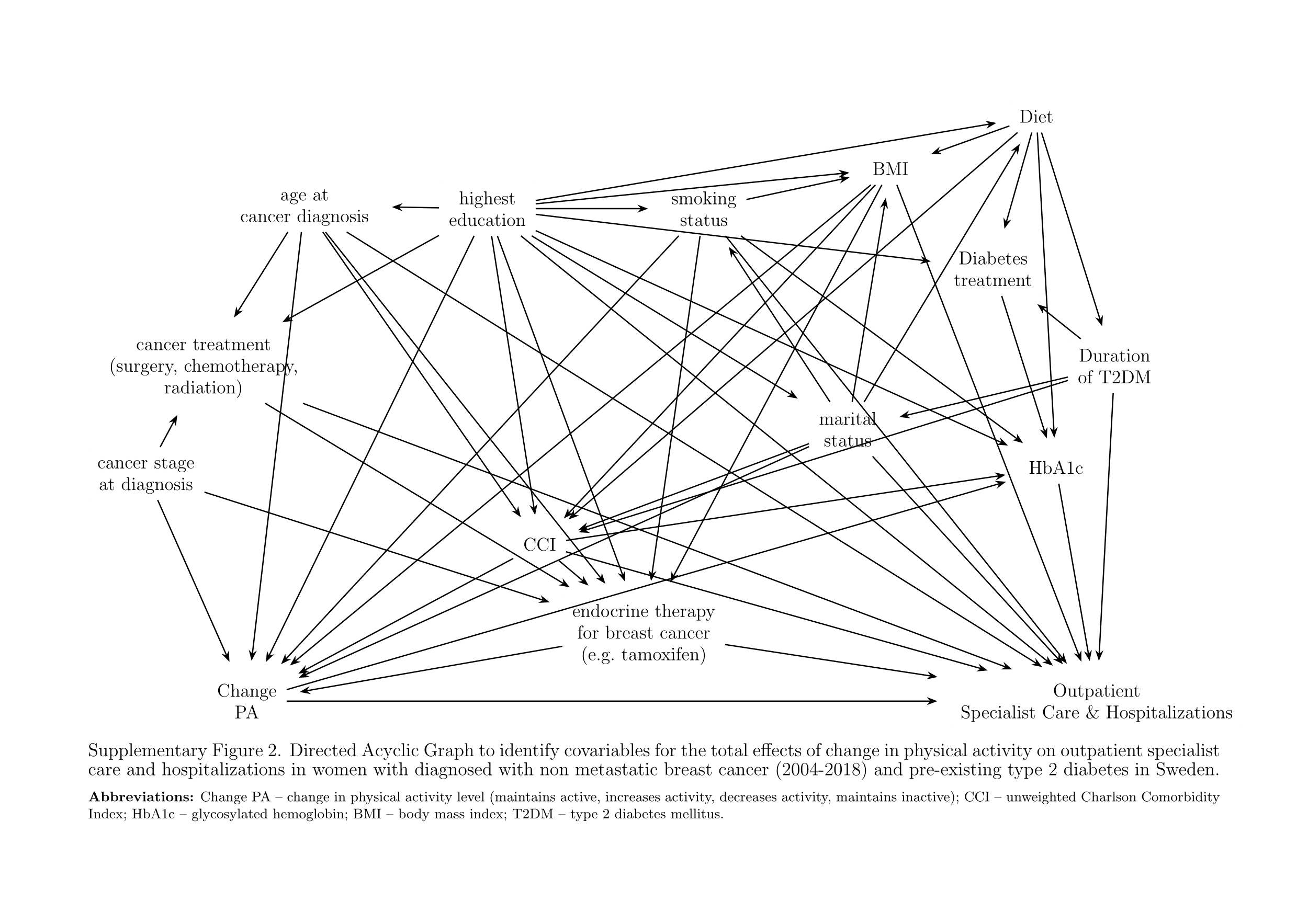


**
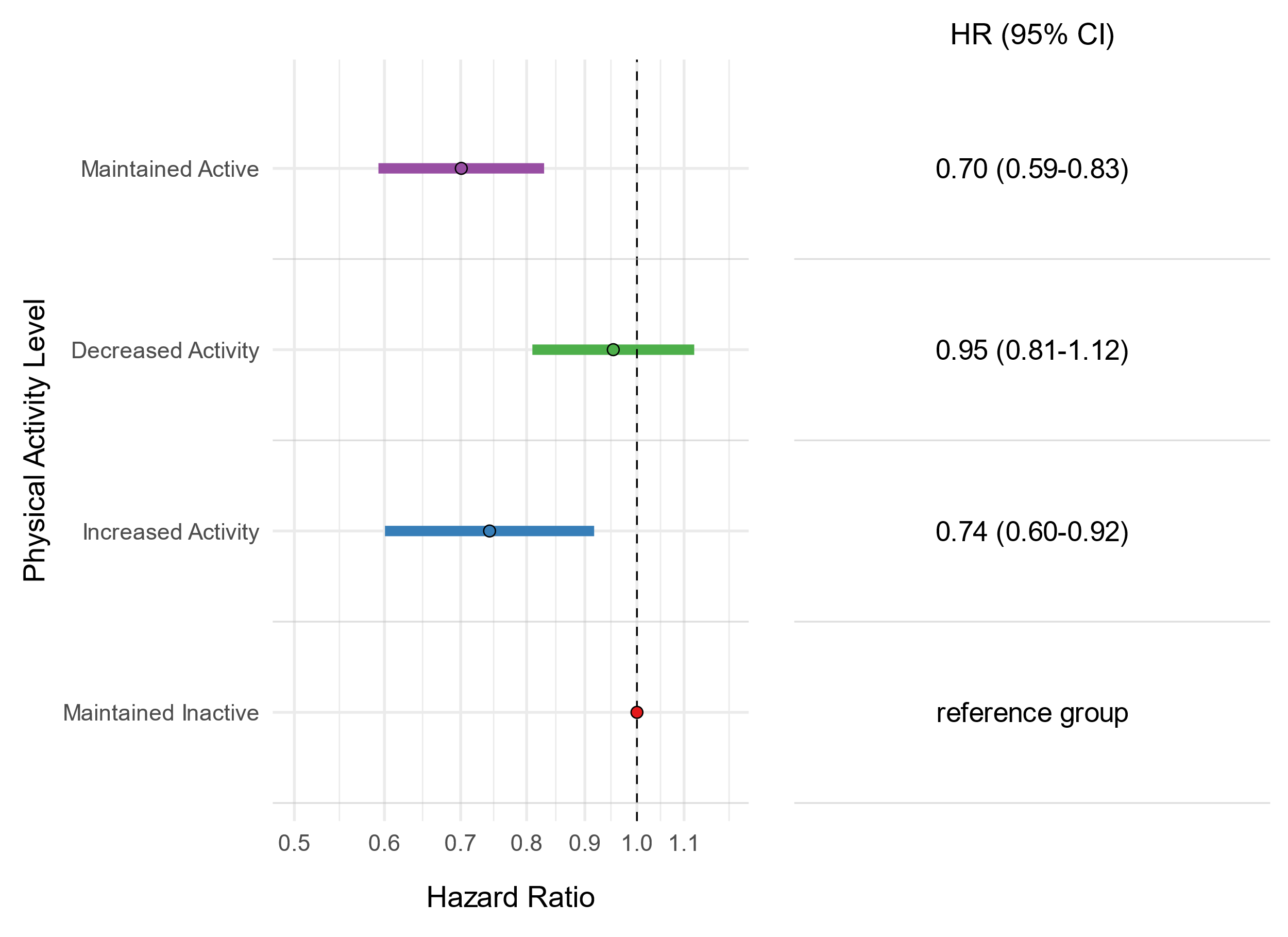
**

**Supplementary Figure 3.** Hazard ratios with 95% confidence interval for hazard of experiencing a hospitalization during follow-up for women 55 years and older (n=2016) with incident, nonmetastatic breast cancer diagnosed between 2004 and 2018 and pre-existing type 2 diabetes. Cox proportional hazard model was adjusted for smoking status, body mass index, age at breast cancer diagnosis, tumor stage, node stage, highest education, marital status, endocrine treatment and unweighted Charlson Comorbidity Index.

Abbreviations: HR – Hazard ratio; CI – Confidence Interval

**
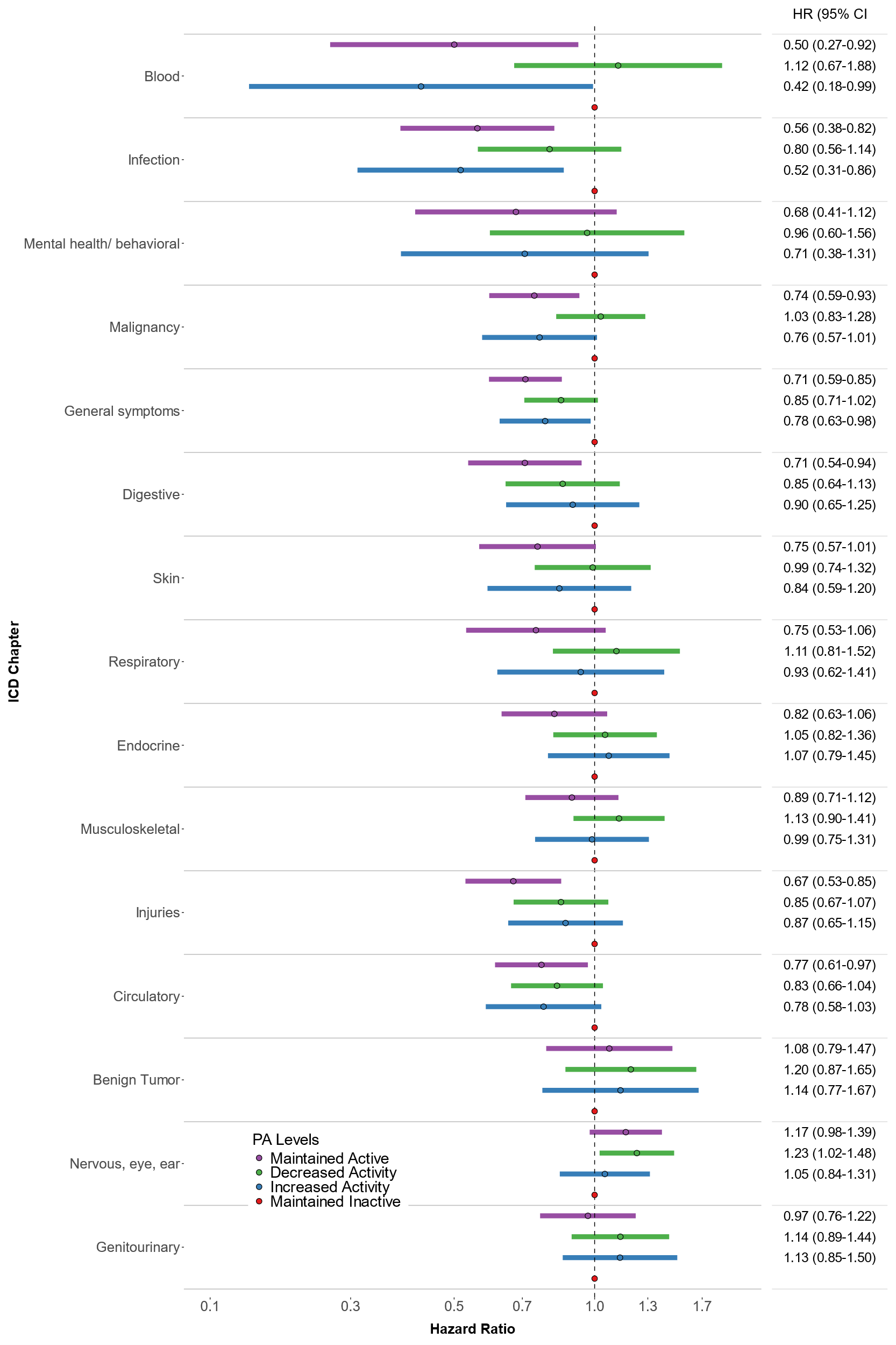
**

**Supplementary Figure 4.** Hazard ratios with 95% confidence interval for first occurance outpatient specialist care or hospitalization by International Classification of Disease (ICD)-10 chapter for women 55 years and older (n=2016) with incident, nonmetastatic breast cancer diagnosed between 2004 and 2018 and pre-existing type 2 diabetes. Cox proportional hazard models were adjusted for smoking status, body mass index, age at breast cancer diagnosis, tumor stage, node stage, highest education, marital status, endocrine treatment and unweighted Charlson Comorbidity Index.

Abbreviations: PA Levels – change in physical activity; HR – Hazard ratio; CI – Confidence Interval

**^
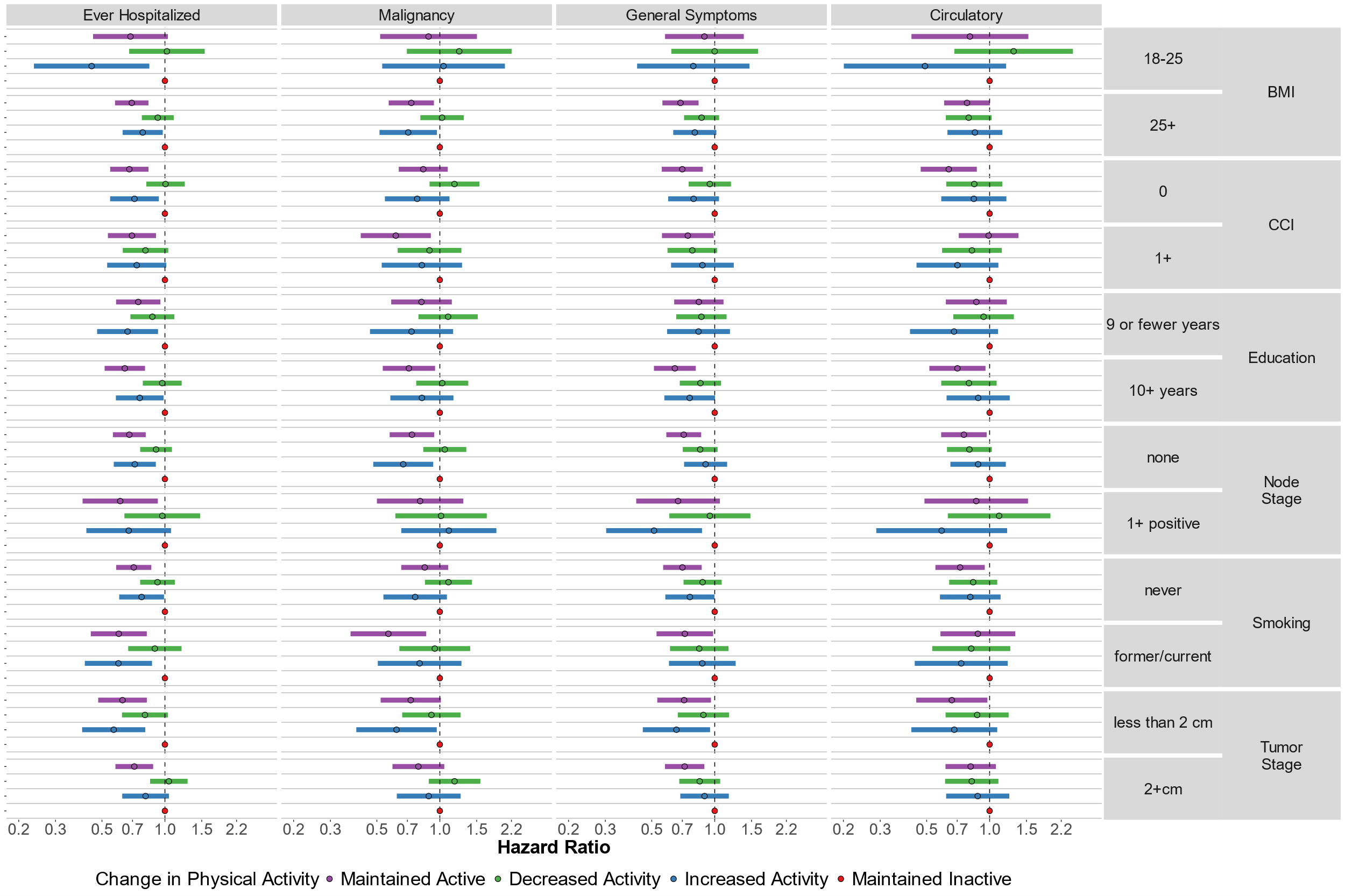
^**

**Supplementary Figure 5**. Hazard ratios and 95% confidence interval for experiencing a hospitalization during follow-up and first-occurrence specialist outpatient care or hospitalization for selected International Classification of Disease-10 chapters, stratified by participant characteristics among 2145 women with incident, nonmetastatic breast cancer diagnosed between 2004 and 2018 and pre-existing diabetes. Cox proportional hazard models were adjusted for smoking status, BMI, age at breast cancer diagnosis, tumor stage, node stage, highest education, marital status, endocrine treatment and CCI, even when stratified.

Abbreviations: PA Levels – change in physical activity; BMI – Body mass index; CCI – unweighted Charlson comorbidity index
